# Effect of Video-Based versus Personalized Instruction on Errors during Elastic Tubing Exercises for Musculoskeletal Pain: A Randomized Controlled Trial

**DOI:** 10.1155/2014/790937

**Published:** 2014-03-10

**Authors:** Kenneth Jay, m. c. schraefel, Mikkel Brandt, Lars L. Andersen

**Affiliations:** ^1^National Research Centre for the Working Environment, Lersø Parkalle 105, 2100 Copenhagen Ø, Denmark; ^2^Electronics and Computer Science, Faculty of Physical and Applied Sciences, University of Southampton, Southampton SO17 1BJ, UK; ^3^Institute of Sports Science and Clinical Biomechanics, University of Southern Denmark, Campusvej 55, 5230 Odense, Denmark

## Abstract

Workplace interventions have shown beneficial results of resistance training for chronic pain in the neck, shoulder, and arm. However, studies have relied on experienced exercise instructors, which may not be an available resource at most workplaces. The objective of this study is to evaluate the technical performance level of upper limb rehabilitation exercises following video-based versus personalized exercise instruction. We recruited 38 laboratory technicians and office workers with neck/shoulder pain for a two-week exercise training period receiving either (1) personal and video or (2) video only instruction in four typical neck/shoulder/arm rehabilitation exercises using elastic tubing. At a 2-week follow-up, the participants' technical execution was assessed by two blinded physical therapists using a reliable error assessment tool. The error assessment was based on ordinal deviation of joint position from the ideal position of the shoulder, elbow, and wrist in a single plane by visual observation. Of the four exercises only unilateral shoulder external rotation had a higher normalized error score in the V group of 22.19 (9.30) to 12.64 (6.94) in the P group (*P* = 0.002). For the remaining three exercises the normalized error score did not differ. In conclusion, when instructing simple exercises to reduce musculoskeletal pain the use of video material is a cost-effective solution that can be implemented easily in corporations with challenging work schedules not allowing for a fixed time of day to go see a personal trainer.

## 1. Introduction

Neck/shoulder pain is a common musculoskeletal complaint in the working population [1]. Repetitive movement tasks requiring sustained low force muscular contractions such as computer and laboratory work impose substantial physical strain on the body that, for many, results in musculoskeletal disorders, such as myalgia of the neck and shoulder, leading to an increase in nociceptive signaling and eventually, for many, becomes painful [2].

As a possible intervention strategy to counter the negative effects of monotonous repetitive work, physical exercise has been introduced as a possible treatment modality based on more than a decade of research in resistance exercise at work with elastic tubing, dumbbells, and kettlebells; there is substantial evidence supporting the effectiveness of brief intensive exercise bouts on musculoskeletal pain and discomfort [3–7]. As little as 2 min of specific shoulder exercises with elastic tubing on a daily basis performing 1 set to failure, has been shown to decrease neck and shoulder pain by a third (on a Visual Analog Pain scale 0–10) while being just as effective as 12 min of 5-6 sets [4, 6]. However, integrating exercise at the worksite can also be costly. If a company is faced with having to hire an exercise instructor or a company's organization does not support opportunities for a fixed training time, the solution may be to have employees use instructional material on their own that has been prefabricated to suit their workday.

The use of prerecorded online accessible video sequences with audible exercise instructions in a bullet point format might be a sustainable solution to the problem. In other domains, video instructional sequences are already being used to teach trainees how to perform even complicated movement tasks requiring fine and gross motor control [8–13] but to our knowledge video instructional material has yet to be investigated as a viable strategy in an exercise-at-work setting.

One challenge with using video-based instructional material for resistance training as an intervention strategy is the anticipated risk of an increase in errors made during exercise when there is no instructor to provide appropriate corrections. Wrongly executed strengthening exercises may result in sprains, strains, tendonitis, bursitis, or impingement of joints and ligaments as well as muscle contusions and general overuse injuries [14, 15]. For instance, the impingement of the subacromial bursa lying between the coracoacromial ligament and the supraspinatus muscle is a common exercise-induced problem and usually occurs with overuse and/or lack of scapulae-humeral rhythm during shoulder abduction movements [14–17]. The main objective of this study, therefore, is to evaluate the technical performance level of four different shoulder, arm, and hand specific exercises using elastic tubing when using either in person or video-based exercise instruction. We hypothesize a higher error score among the participants receiving video-based instructional material compared with personal instruction.

## 2. Methods

### 2.1. Study Design

In this assessor-blinded randomized controlled trial, we recruited 38 participants (laboratory technicians and office workers) from a pool of 200 people at a large pharmaceutical company in Copenhagen, Denmark. To be eligible to participate in the study the inclusion criteria were (1) a history of neck or shoulder pain during the previous week with an intensity of at least 2 on a scale of 0–10, (2) female aged 18–67 years, and (3) no prior experience exercising with elastic tubing. Exclusion criteria were (1) blood pressure higher than 160/100, (2) pregnancy, and (3) life-threatening disease or other adverse health conditions and contraindications towards resistance exercise. The participants were recruited based on their answers to a recruitment-screening questionnaire. The included participants were randomly allocated using concealed envelopes to either a personal + video instruction (P) group (*n* = 19) or a video-based instruction (V) group (*n* = 19). Following two weeks of training four different shoulder, arm, and hand exercises with elastic tubing, the participants were invited in for an error assessment evaluation of the technical exercise execution.

### 2.2. Participants

All participants (*n* = 38) were informed about the main objective and content of the project and gave written informed consent to participate in the study, which conformed to the Declaration of Helsinki. The study was approved by the Local Ethical Committee (H-3-2010-062). Concealed random allocation to one of the two groups (“P” or “V”) was performed. Baseline demographics after group allocation with descriptive statistics before and after training are shown in Table 1.

### 2.3. Dropouts

200 people received an informational email about the study. 49 people agreed to answer a baseline-screening questionnaire and 38 were invited to participate. One person was excluded due to lack of answering the screening questionnaire and four people did not show up for the second error assessment by the examiners due to sickness unrelated to the study.

### 2.4. Exercise Error Assessment

**Figure 1:**
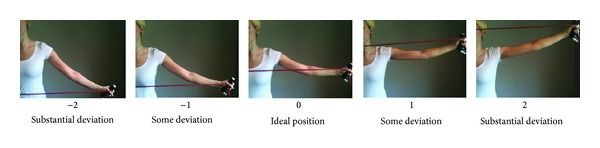
The possible error positions to either side from the ideal position in the horizontal plane of the bilateral shoulder retraction exercise. Pictures show the left side. Negative deviations were denoted by angles below 90 degrees of the joint and positive deviations were applied to angles above 90 degrees.

Two physical therapists assessed the number of errors following two weeks of training in four common shoulder, arm, and hand exercises using elastic tubing and have been described in detail previously (Jay et al., the correct issue where the reliability article appears). In short, the four exercises were as follws: (a) bilateral raise, (b) Bilateral scapular retraction, (c) unilateral external shoulder rotation, and (d) unilateral wrist extension. Each exercise was described by joint (wrist, elbow, and shoulder) and ordinal deviation from the ideal position in a single plane, by visual observation. For each joint the examiners had to evaluate by how much the position of the joint deviated from the ideal position, as well as to what side from ideal, during exercise execution. The possible deviations were denoted as “no deviation,” “some deviation,” or “substantial deviation.” Negative deviations were denoted for angles below 90 degrees of the joint and positive deviations were applied to angles above 90 degrees and had fair to substantial intra- and intertester reliability, respectively [18]. The examiners were kept blinded and instructed not to provide any feedback to the participants on the execution of each exercise. Each participant performed 2 sets of 10 repetitions during technical exercise execution evaluation. One set was performed in front view and one set was performed showing a side view [18]. Furthermore each participant was instructed not to reveal if they had been receiving P or V instruction. The same examiner assessed each participant twice with at least one day in between.  Figure 1 provides an example of how the deviation was noted.

### 2.5. Video-Based Exercise Instruction

The video-based exercise instruction consisted of four short videos showing a person properly performing the exercises. Audio instructions of general guidelines for each exercise, as well as for areas of special attention, were dubbed over the videos. The instructions included setting up the exercise (i.e., positioning and anchoring of the elastic tubing) and the correct shoulder, wrist, and hand position during the exercise, as well as exercise tempo and number of repetitions. The model in the video was shown in full figure from one angle completing a full set of repetitions. Furthermore, the participants allocated to this group were also given a set of written instructions with pictures of each of the four exercises. Videos and written instructions were emailed to each participant for ease of accessibility and a set of elastic tubing was handed out at the day of the concealed-envelope randomization. Furthermore, the V group was encouraged to exercise as frequently as possible using the provided instructions during company working time as well as in their leisure time. Online assessable instructional material was made available at http://www.jobogkrop.dk/Ondt-i-muskler-og-led/Ondt-i-nakke-skulder-og-arm/Elastikoevelser-for-nakke-skulder-og-arm.

### 2.6. Personalized Exercise Instruction

The participants randomized to the personalized instruction group were provided with the same instructional material as the participants in the V group with the addition of having the possibility of receiving personalized exercise instruction and correction by an experienced trainer for sessions of 10 min 5 days per week at the worksite between 9 a.m. and noon. The participants in this group were allowed to attend the supervised training sessions during company working hours with no limit to how frequent they could attend on a daily basis. Finally, like the participants of group V, the P group was encouraged to exercise as frequently as possible during working hours and at their own time. Table 1 summarizes descriptive statistics including training frequency. A set of elastic tubing as provided for them to take home.

### 2.7. Outcomes

The primary outcome of this study is errors in exercise execution following the two types of instructional training (P or V, resp.). We also report descriptive statistics on training frequency, use of written and video instructional material, and personalized training adherence as well as pre- to posttraining self-perceived pain of the neck, shoulder, arm, and wrist (Table 1).

### 2.8. Statistics

Variables were analyzed in accordance with the CONSORT statement for randomized controlled trials intention-to-treat principle; that is, dropouts from the two-week training were invited to participate in the error assessment to avoid selection bias. Differences were determined by performing analysis of variance (Proc Mixed) with the appropriate post hoc testing of the SAS statistical software (SAS institute, Cary, NC, version 9.2). We accept *P* < 0.05 as statistically significant and report results as an averaged normalized error score (0–100) of the two assessment rounds with the two examiners' mean (SD) and 95% confidence intervals (95% CI) where appropriate.

## 3. Results

Table 1 shows demographics, adherence to both training interventions, and changes in pain. The number of training sessions was 8.8 and 7.3 out of 10 during the two weeks in the P and V groups, respectively. The average use of video and written material was 0.26 and 5.53 times for the P group and 2.42 and 3.74 times for the V group, respectively. The average decrease in pain for neck, shoulder, and combined elbow, wrist, and forearm in the P group was 35%, 30%, and 50%, respectively. For the V group similar results were found. For neck, shoulder, and combined elbow, wrist, and forearm the average decrease in pain was 41%, 48%, and 39%, respectively.

Table 2 summarizes the error scores of the two groups. Of the four exercises only unilateral shoulder external rotation had a higher normalized error score in the V group of 22.19 (9.30) to 12.64 (6.94) in the P group (*P* = 0.002). For the remaining exercises the normalized error score did not differ (*P* > 0.05). Post hoc analysis revealed that two of the six subdomains of unilateral shoulder external rotation were significantly different in group V compared to group P. Those domains were as follows: (1) flexion of the elbow −0.51 (0.33) (*P* < 0.001) and (2) abduction of the shoulder joint 0.27 (0.36) (*P* < 0.03).  Table 3 summarizes post hoc tests for all of the subdomains in each of the four exercises.

## 4. Discussion

To our knowledge, this is the first time a study has investigated the error score of technical exercise execution during resistance exercises comparing personalized instruction with video based instruction. In contrast to our hypothesis, the error scores were not significantly higher in the video-group in three of the four exercises. Only unilateral shoulder external rotation differed significantly, showing a higher normalized error score in the V group of 22.19 (9.30) to 12.64 (6.94) in the P group (*P* = 0.002) (Table 2). Analysing the post hoc test results showed that the subdomains of the exercise that differed were amount of elbow flexion (*P* < 0.001) and shoulder abduction (*P* < 0.03) (Table 3); that is, the elbow was more extended and the shoulder more abducted in the video group. Conversely, the technical execution of unilateral wrist extension tended to be better in the V group compared to the P group with an error score of approximately 34 to 46, respectively (*P* = 0.07) (Table 2).

Surprisingly, our hypothesis of a higher error score in the V group was not verified in three of the four exercises, which demonstrates that a visual input of the movement is an important factor in motor learning and can possibly compensate for lack of kinaesthetic feedback from a trainer/coach. In most instructional situations where motor skills are to be learned, performers are given instructions about the correct movement pattern, which typically refer to specific body segments in relation to timing, position, and trajectory [19]. According to Wulf [19] this creates an internally driven focus, which has repeatedly been shown to be an inefficient way of acquiring new movement skills [20–24]. Instead Wulf suggests an external target-driven cueing approach where the trainee focuses on either moving the implement (as opposed to the limbs holding the implement) or the movement trajectory to be performed [19]. This suggests that, in our study, the V group, when watching the instructional videos, may have been more focused on making the exercise movement look like the movement in the video as opposed to concentrating on keeping the individual joints in the right position. For the one exercise (unilateral shoulder external rotation) having a higher error score in the V group may be related to lack of visual information from the video combined with the actual limb movement being a kinaesthetic challenge for most untrained people as the elbow of the working arm has to stay flexed to about 90 degrees in a fixed position, while the humerus rotates out and in along its longitudinal axis. It could be speculated that if the exercise had been shown from multiple angles in the video, the V group may have become aware of the elbow position thereby reducing the error score subdomains of elbow flexion and shoulder abduction.

In human-computer interaction [25] the external focus model using environment markers in motor learning suggests that by using a perception camera (Kinect) to detect movements while creating a virtual-reality environment [26] with an avatar replicating the person's movements standing next to a “teacher” avatar showing the “correct” movement while providing verbal feedback is an innovative idea that combines the external attentional focus with recent advances in computer technology. The present study suggests that using visual feedback can indeed be just as effective as having an instructor present when learning simple movement tasks and the work currently being done experimentally in human-computer interactions may represent the next step in teaching exercises at the workplace.

Limitations to the present study include the lack of objective assessment measures, for example, joint angle kinematics, to validate the examiners' observations and the limited number of participants in each group. Strengths of the present study include the assessor-blind randomized controlled study design and the simple assessment protocol requiring no tools or technical equipment [18]. Our work also demonstrates the viability of combining video models with affordable movement tracking for “virtual trainers.” Such systems can be designed to respond to participant error and offer the kinds of corrections seen in our study that improve performance of less familiar movements. In the meantime, however, we see that video instruction on its own has strong practical efficacy. Furthermore, King et al. have shown that adherence to and long term maintenance of exercise programs without a personal trainer are possible by simple self-monitoring strategies [27].

In conclusion, when instructing simple exercises to reduce musculoskeletal pain and discomfort, the use of video material is a robust solution that can be easily implemented in corporations with challenging work schedules that may not allow for a fixed time of day to go see a personal trainer. Furthermore video delivery is a cost-effective way to integrate exercise at work.

## Figures and Tables

**Table 1:** Participant baseline demographics and descriptive statistics mean (SD) following concealed-envelope randomization to one of the two exercise groups.

	P-group mean (SD)	V-group mean (SD)
Baseline demographics		
Number of participants	19	19
Age (years)	47 (10)	43 (6)
Weight (kg)	66.1 (10.0)	70.3 (8.6)
Height (cm)	168.7 (7.0)	171.1 (6.8)
Descriptive statistics		
Pain (mVAS 0–10)	Pre/post training	Pre/post training
Neck	4.79 (2.3)/3.11 (2.35)	5.37 (2.63)/3.16 (2.03)
Shoulder	4.63 (2.43)/3.32 (2.31)	4.95 (3.29)/2.58 (2.41)
Elbow, forearm, and wrist/hands	3.16 (2.65)/1.58 (1.64)	2.68 (2.85)/1.63 (2.36)
Adherence		
Training frequency	8.79 (2.18)	7.26 (3.00)
Use of written material for reference	5.53 (3.45)	3.74 (2.60)
Use of video material for reference	0.26 (0.56)	2.42 (1.74)
Number of times receiving personalized instruction	5.63 (2.14)	0.05 (0.23)

**Table 2:** Results of Proc Mixed procedure for normalized error score of the four exercises for the two groups mean (SD).

Exercise	Normalized error score (0–100)		
	*N*	Mean (SD)	*P* value
Bilateral raise			
P	18	23.78 (10.66)	0.75
V	17	25.11 (13.50)	
Bilateral scapular retraction			
P	18	13.40 (8.42)	0.63
V	17	15.22 (13.28)	
Unilateral shoulder external rotation			
P	18	12.64 (6.94)	0.002*
V	16	22.19 (9.30)	
Unilateral wrist extension			
P	18	45.83 (21.05)	0.07
V	17	33.82 (16.79)	

*Indicates significant difference (*P* < 0.05) between the two groups. For the shoulder external rotation exercise the normalized error score is significantly higher in V group compared to P group.

**Table 3:** Normalized error score of the subdomains of each exercise mean (SD) and 95% CI.

Exercise	Joint	Subdomain (left/right)	Exercise error assessment					
			P group		V group		Group difference (P − V)	
			Mean (SD)	95% CI	Mean (SD)	95% CI	Mean (SD)	95% CI
Bilateral raise	W	*Palmar/dorsal flexion (L) *	0.48 (0.62)	(0.16 : 0.81)	0.13 (0.52)	*(−0.14 : 0.39) *	0.36 (0.57)	(−0.04 : 0.76)
	W	*Palmar/dorsal flexion (R) *	0.56 (0.48)	(0.33 : 0.80)	0.10 (0.72)	*(−0.26 : 0.46) *	0.47 (0.61)*	(0.05 : 0.88)
	W	*Radial/ulnar deviation (L) *	0.13 (0.30)	(−0.04 : 0.29)	0.18 (0.27)	*(0.05 : 0.31) *	−0.06 (0.29)	(−0.26 : 0.14)
	W	*Radial/ulnar deviation (R) *	0.10 (0.26)	(−0.03 : 0.23)	0.15 (0.23)	*(0.04 : 0.27) *	−0.06 (0.24)	(−0.22 : 0.11)
	E	*Elbow flexion (L) *	0.08 (0.25)	(−0.06 : 0.21)	−0.08 (0.32)	*(−0.24 : 0.08) *	0.16 (0.29)	(−0.04 : 0.37)
	E	*Elbow flexion (R) *	0.01 (0.18)	(−0.08 : 0.10)	−0.10 (0.31)	*(−0.25 : 0.06) *	0.11 (Satterthwaite)	(−0.06 : 0.29)
	S	*Horizontal plane position (V) *	0.33 (0.35)	(0.14 : 0.51)	−0.24 (0.34)	*(−0.40 : −0.07) *	0.56 (0.34)*	(0.32 : 0.80)
	S	*Horizontal plane position (R) *	0.35 (0.33)	(0.18 : 0.51)	−0.24 (0.34)	*(−0.40 : −0.07) *	0.58 (0.36)*	(0.36 : 0.81)
	S	*Adduction/abduction (L) *	0.44 (0.38)	(0.23 : 0.64)	0.53 (0.42)	*(0.32 : 0.74) *	−0.09 (0.40)	(−0.37 : 0.19)
	S	*Adduction/abduction (R) *	0.46 (0.37)	(0.28 : 0.64)	0.53 (0.42)	*(0.32 : 0.74) *	−0.07 (0.39)	(−0.34 : 0.20)
	S	*Humeral rotation (L) *	0.19 (0.23)	(0.06 : 0.31)	0.10 (0.26)	*(−0.03 : 0.23) *	0.09 (0.25)	(−0.08 : 0.26)
	S	*Humeral rotation (R) *	0.17 (0.23)	(0.05 : 0.28)	0.10 (0.26)	*(−0.03 : 0.23) *	0.07 (0.24)	(−0.09 : 0.23)

Bilateral shoulder retraction	W	*Palmar/dorsal flexion (L) *	−0.22 (0.65)	(−0.54 : 0.10)	−0.10 (0.71)	*(−0.45 : 0.25) *	−0.13 (0.68)	(−0.58 : 0.33)
	W	*Palmar/dorsal flexion (R) *	−0.22 (0.69)	(−0.56 : 0.12)	−0.17 (0.60)	*(−0.47 : 0.13) *	−0.06 (0.64)	(−0.49 : 0.38)
	W	*Radial/ulnar deviation (L) *	−0.03 (0.08)	(−0.07 : 0.01)	−0.10 (0.33)	*(−0.26 : 0.07) *	0.07 (Satterthwaite)	(−0.10 : 0.25)
	W	*Radial/ulnar deviation (R) *	−0.01 (0.06)	(−0.04 : 0.02)	−0.08 (0.27)	*(−0.22 : 0.05) *	0.07 (Satterthwaite)	(−0.06 : 0.21)
	E	*Elbow flexion (L) *	0.10 (0.23)	(−0.02 : 0.21)	0.13 (0.27)	*(−0.01 : 0.26) *	−0.03 (0.25)	(−0.20 : 0.14)
	E	*Elbow flexion (R) *	0.07 (0.21)	(−0.03 : 0.17)	0.10 (0.31)	*(−0.06 : 0.25) *	−0.03 (0.26)	(−0.21 : 0.15)
	S	*Horizontal plane position (L) *	0.36 (0.58)	(0.07 : 0.65)	0.38 (0.51)	*(0.12 : 0.63) *	−0.01 (0.55)	(−0.39 : 0.36)
	S	*Horizontal plane position (R) *	0.36 (0.58)	(0.07 : 0.65)	0.38 (0.51)	*(0.12 : 0.63) *	−0.01 (0.55)	(−0.39 : 0.36)
	S	*Adduction/abduction (L) *	0.22 (0.37)	(0.04 : 0.41)	−0.01 (0.13)	*(−0.08 : 0.05) *	0.27 (Satterthwaite)*	(0.04 : 0.43)
	S	*Adduction/abduction (R) *	0.22 (0.37)	(0.04 : 0.41)	−0.01 (0.13)	*(−0.08 : 0.05) *	0.24 (Satterthwaite)*	(0.04 : 0.43)
	S	*Humeral rotation (L) *	0.00 (0.09)	(−0.04 : 0.04)	−0.38 (0.60)	*(−0.67 : −0.08) *	0.38 (Satterthwaite)*	(0.07 : 0.68)
	S	*Humeral rotation (R) *	0.00 (0.09)	(−0.04 : 0.04)	−0.29 (0.54)	*(−0.56 : −0.02) *	0.29 (Satterthwaite)*	(0.02 : 0.56)

Unilateral shoulder external rotation	W	*Palmar/dorsal flexion (R) *	−0.78 (0.56)	(−1.05 : −0.50)	−0.85 (0.60)	*(−1.16 : −0.54) *	0.08 (0.58)	(−0.32 : 0.47)
	W	*Radial/ulnar deviation (R) *	0.01 (0.06)	(−0.02 : 0.04)	0.09 (0.26)	*(−0.05 : 0.22) *	−0.07 (Satterthwaite)	(−0.21 : 0.06)
	E	*Elbow rotation (R) *	−0.01 (0.06)	(−0.04 : 0.02)	0.00 (0.00)	*(0.00 : 0.00) *	−0.01 (Satterthwaite)	(−0.04 : 0.02)
	E	*Elbow flexion (R) *	0.11 (0.27)	(−0.03 : 0.25)	0.62 (0.38)	*(0.42 : 0.81) *	−0.51 (0.33)*	(−0.73 : −0.28)
	S	*Shoulder flexion (R) *	−0.06 (0.20)	(−0.16 : 0.05)	−0.04 (0.42)	*(−0.26 : 0.17) *	−0.01 (Satterthwaite)	(−0.24 : 0.22)
	S	*Adduction/abduction (R) *	0.13 (0.29)	(−0.02 : 0.27)	−0.15 (0.42)	*(−0.37 : 0.07) *	0.27 (0.36)*	(0.02 : 0.52)

Unilateral wrist extension	W	*Palmar flexion at bottom pos. (R) *	−0.31 (0.63)	(−0.62 : 0.01)	−0.06 (0.30)	*(−0.21 : 0.10) *	−0.25 (Satterthwaite)	(−0.59 : 0.09)
	W	*Dorsal flexion at top pos. (R) *	0.81 (0.35)	(0.63 : 0.98)	0.82 (0.35)	*(0.64 : 0.99) *	−0.01 (0.35)	(−0.25 : 0.22)
	W	*Radial/ulnar deviation (R) *	−0.04 (0.13)	(−0.11 : 0.02)	−0.06 (0.14)	*(−0.12 : 0.01) *	0.01 (0.13)	(−0.08 : 0.10)

*Indicates significant difference between the two groups in the subdomain of the exercise error assessment. Satterthwaite refers to unequal variances in the comparison of the subdomain.
